# Traumatic C1–2 posterolateral dislocation with dens fracture, injury of the transverse atlantal ligament, and unilateral facet fracture with subluxation of C6–7: A case report

**DOI:** 10.1097/MD.0000000000008913

**Published:** 2017-12-01

**Authors:** Jong-Beom Park, Sung Shik Kang, Jin S. Yeom

**Affiliations:** a Department of Orthopaedic Surgery, Uijeongbu St. Mary's Hospital, The Catholic University of Korea, Uijeongbu; b Department of Orthopaedic Surgery and Spine Center, Seoul National University Bundang Hospital, Sungnam, Korea.

**Keywords:** C1–2 dislocation, dens fracture, rupture of transverse atlantal ligament, subaxial cervical injury

## Abstract

**Rationale:**

Traumatic C1–2 dislocation associated with contiguous or noncontiguous cervical spine injury is rare. Moreover, there have been no reports describing traumatic C1–2 dislocation associated with multiple contiguous and noncontiguous cervical injuries.

**Patient concerns:**

The authors present a case of a 20-year-old male with painful limitation of motion of the neck. This complex cervical injury occurred due to hyperextension of the head in a rotated position. The patient complained of neck pain that radiated to the left shoulder and arm, but he did not exhibit any neurological abnormalities.

**Diagnoses:**

The diagnosis of the patients was traumatic C1-2 posterolateral dislocation associated with type II dens fracture (Anderson and D’Alonzo classification), type II injury of the transverse atlantal ligament (Dickman classification), and unilateral facet fracture with subluxation of C6–7.

**Interventions:**

The C1–2 posterolateral dislocation with type II dens fracture was successfully reduced by skull traction. The patient underwent anterior discectomy, open reduction, and fusion with plate fixation of C6–7 followed by posterior segmental fixation and fusion of C1–2.

**Outcomes:**

At his postoperative 1-year follow-up, solid fusion was noted with improvement of clinical symptoms. This is the first report of traumatic C1–2 posterolateral dislocation associated with multiple C2 injuries and noncontiguous subaxial cervical injury.

**Lessons:**

A high index of suspicion and careful evaluation of entire cervical spine should be considered as the key to the proper diagnosis and treatment of traumatic C1–2 dislocation associated with contiguous and noncontiguous cervical injuries.

## 1 Introduction

Traumatic C1–2 dislocation associated with contiguous or noncontiguous cervical spine injury is rare. To date, only a few cases have been reported in the English literature.^[1–7]^ Moreover, there have been no reports describing traumatic C1–2 dislocation associated with multiple contiguous and noncontiguous cervical injuries. Here, we report the first case of traumatic C1–2 posterolateral dislocation associated with type II dens fracture (Anderson and D’Alonzo classification),^[8]^ type II injury of the transverse atlantal ligament (TAL) (Dickman classification),^[9]^ and noncontiguous unilateral facet fracture with subluxation of C6–7.

## 2 Case report

**Figure 1:**
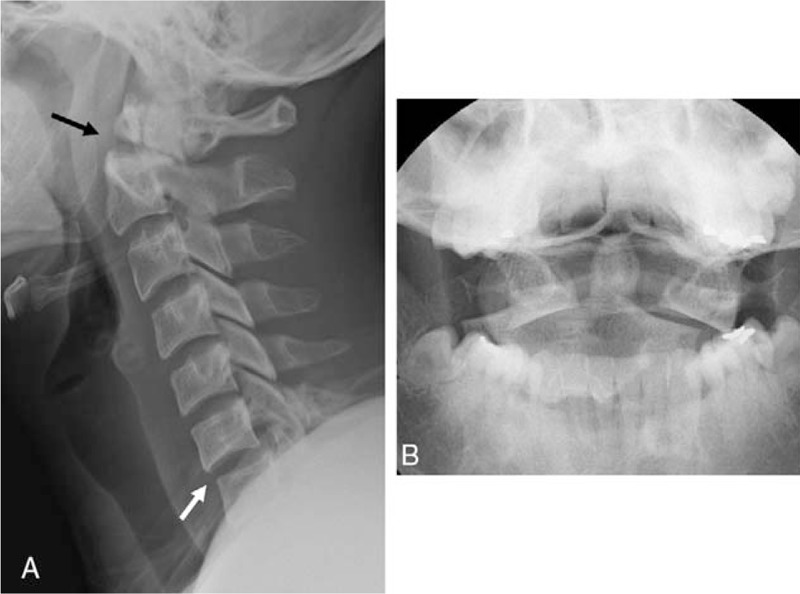
Lateral radiograph (A) and open mouth view (B) showing a posterolateral dislocation of C1 on C2 with posterolateral displacement of the proximal fragment of the dens (black arrow) (type II fracture of Anderson and D’Alonzo classification) and anterior subluxation of C6–7 (white arrow).

**Figure 2:**
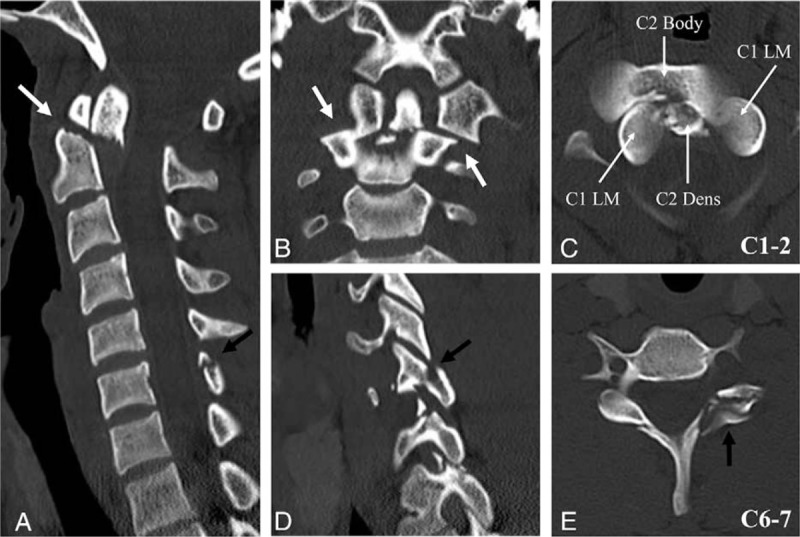
Computed tomography (CT) scans showing posterolateral dislocation of C1 on C2 (white arrows) with type II dens fracture (Anderson and D’Alonzo classification) (A, B, and C), unilateral facet fracture, and subluxation of C6–7 (black arrows) (D and E).

**Figure 3:**
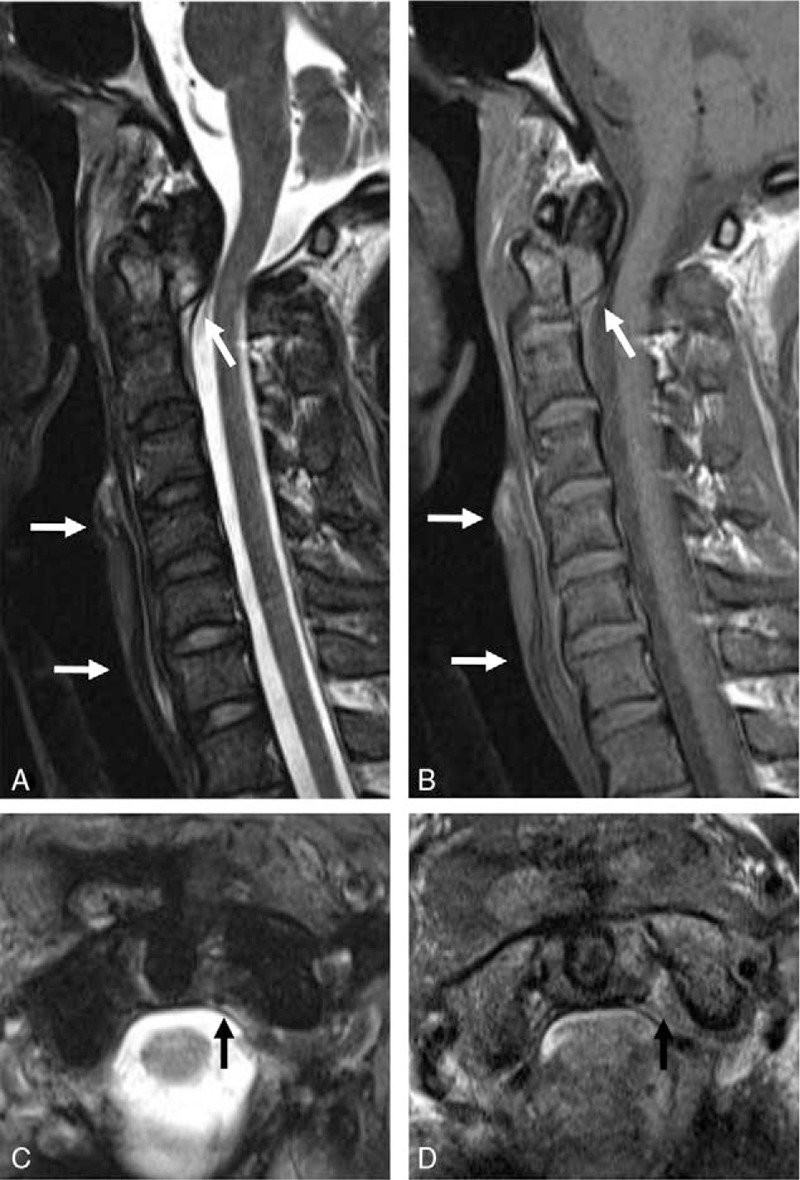
Magnetic resonance (MR) images showing detachment of the posterior longitudinal ligament from the posterior aspect of the dens (white arrows) (A and B) and type II injury of the transverse atlantal ligament (black arrows) (C and D). Acute hemorrhagic fluid collection (white arrows) was seen along the retropharyngeal space of the subaxial spine (A and B).

A 20-year-old male who complained of neck pain that radiated to the left shoulder and arm was admitted to the emergency room. His symptoms developed after accidentally hitting his head on a horizontal bar while riding a bicycle with his head in a rotated position. However, physical examination revealed no specific neurological abnormalities. Plain radiographs of the cervical spine showed traumatic C1–2 posterolateral dislocation associated with type II dens fracture that was established on the basis of the Anderson and D’Alonzo classification and anterior subluxation of C6–7 (Fig. 1). Computed tomography (CT) scans confirmed the dislocation with type II dens fracture (Anderson and D’Alonzo classification)^[2]^ and revealed a unilateral facet fracture with subluxation and lamina fracture of C6–7 (Fig. 2). Magnetic resonance (MR) images showed a detached posterior longitudinal ligament from the posterior aspect of the dens and type II TAL (Dickman classification) injury^[3]^ (Fig. 3). Acute hemorrhagic fluid was seen collecting along the retropharyngeal space of the subaxial spine.

**Figure 4:**
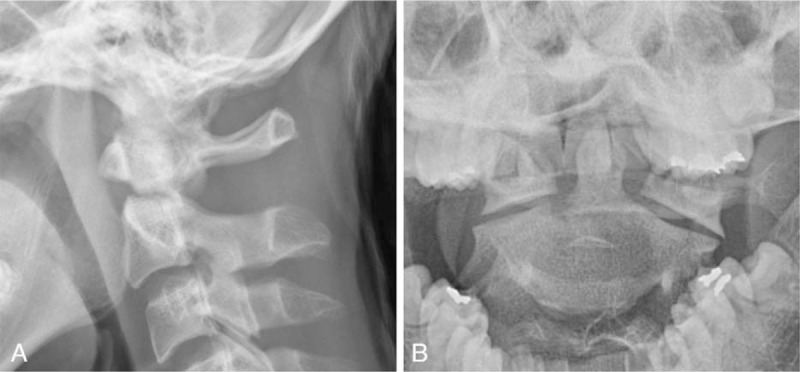
Lateral radiograph (A) and open mouth view (B) showing successful reduction of C1–2 posterolateral dislocation with type II dens fracture by skeletal traction.

**Figure 5:**
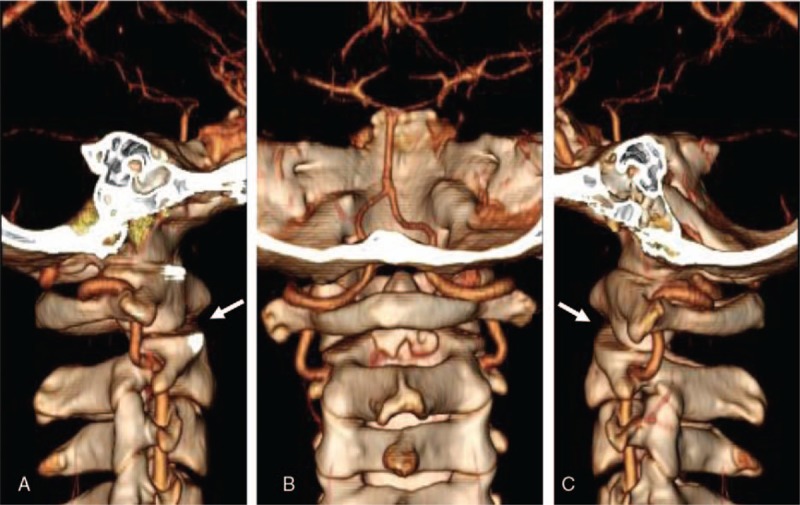
Neck angio-CT scans showing a normal vertebral artery and reduction of C1–2 posterolateral dislocation with type II dens fracture.

**Figure 6:**
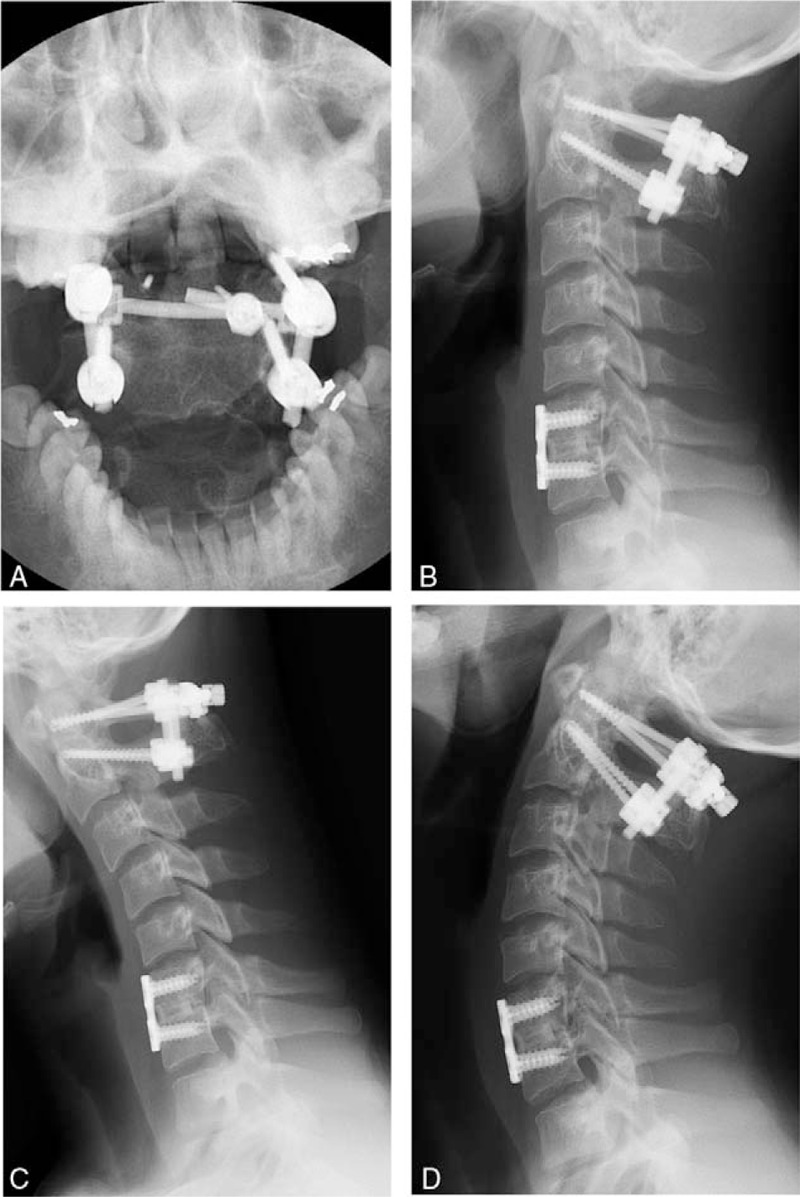
One-year postoperative plain radiographs show solid union of C1–2 and C6–7 without abnormal motion.

**Figure 7:**
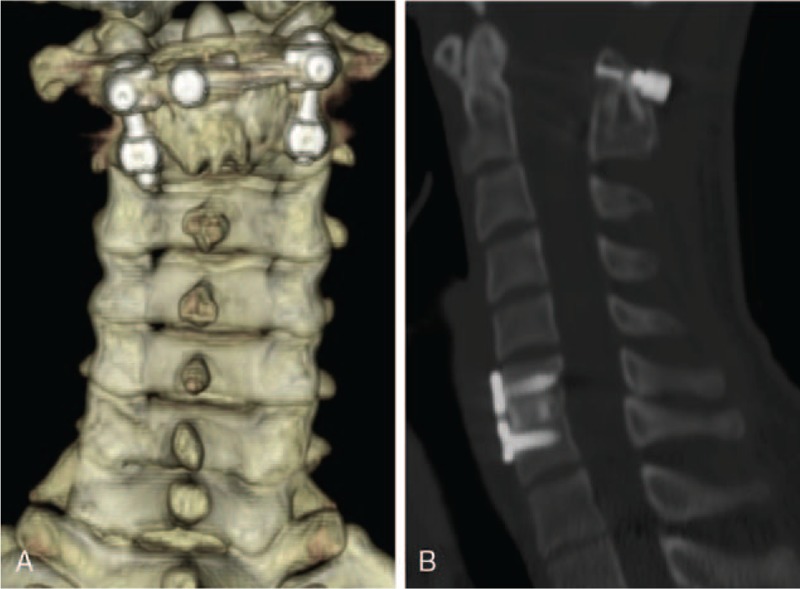
One-year postoperative computed tomography (CT) scans show solid union of C1–2 and C6–7.

Skeletal traction was gently applied using a Gardner well tong. Seven pounds was used as the initial traction weight, and it was gradually increased to 18 pounds under fluoroscopy for 2 days. Closed reduction of the traumatic C1–2 posterolateral dislocation with type II dens fracture was successfully achieved (Fig. 4). After closed reduction, angio-CT scans of the neck showed a normal vertebral artery at the C1–2 level, but some degree of residual subluxation of the C1–2 joint with type II dens fracture (Fig. 5). While maintaining skull traction with 7 pounds, we performed anterior discectomy, open reduction, and fusion of C6–7 with plate fixation (Aspiron, U & I, Uijeongbu, Korea) and allograft (Cortical cervical spacer; DCI Donor Service, Albuquerque) packed with demineralized bone matrix (DBX; Musculoskeletal Transplantation Foundation, Edison). The patient was then placed in the prone position, and posterior segmental screw fixation and fusion of C1–2 were performed. Bilateral C1 posterior arch screws and bilateral C2 pedicle screws (Vertex Select; Medtronic, Memphis) were inserted. The cartilaginous portions of the C1–2 facet joints were thoroughly removed. Meticulous decortication of C1–2, including the facet joints, and sufficient bone grafting with autogenous iliac crest were performed in order to obtain solid fusion. Finally, rods and a crosslink set were applied and tightened. At 2 months after surgery, the patient's neck pain and occipital neuralgia had nearly resolved. One year after surgery, plain radiographs of the cervical spine showed solid union, and dynamic radiographs showed no abnormal motion of the C1–2 and C6–7 segments (Fig. 6). Reconstructed 2-dimensional and 3-dimensional CT scans also confirmed anatomical reduction and solid fusion of the C1–2 and C6–7 segments (Fig. 7).

## 3 Discussion

Traumatic C1–2 dislocation associated with dens fracture is a complex and unstable injury. This type of injury is rarely reported, so a definitive treatment strategy has not been established.^[1–7]^ However, most studies used closed reduction as first-line treatment. After successful closed reduction, the need for C1–2 fusion depends on the integrity of the TAL and the stability of the dens fracture and C1–2 joint. The TAL is one of the most important structures for providing C1–2 stability. If complete injury of the TAL is identified, posterior C1–2 fusion must be performed. In addition, if instability or subluxation of the C1–2 joint persists after closed reduction, posterior C1–2 fusion is mandatory. If the C1–2 dislocation cannot be reduced by closed reduction, open reduction and fusion should be performed. Treatment options for dens fracture, including cervical brace, halovest, anterior screw fixation, and posterior C1–2 fusion, are still controversial^[8,10,11]^ If the displacement is posterior, more than 5 to 6 mm, or more than 10° angulation, there is a high risk of nonunion of the dens fracture. Therefore, the choice of treatment for dens fractures must take into account the location, degree of displacement, and angulation.

Our case report has several features that distinguish it from the previous reports. First, type of our C1–2 injury is posterolateral dislocation without any significant neurologic deficit. Second, associated contiguous C2 injury is multiple consisting of type II dens fracture (Anderson and D’Alonzo classification) and type II TAL injury (Dickman classification). Third, our case is associated with noncontiguous subaxial C6–7 injury treated by surgery. Finally, our case report provided 2- and 3-dimensional reconstructed CT scans and MR images to clearly diagnose this complicated multiple injury.

Our patient's injury was complex and highly unstable because the C1–2 posterolateral dislocation was associated with type II dens fracture and type II TAL injury. Dens fracture and TAL injury are generally caused by axial loading force. In addition, C1–2 joint with dens fracture is posteriorly displaced by extension force.^[1,8,9]^ Our case was a C1–2 posterolateral dislocation with dens fracture and TAL injury. Given that the C1–2 posterolateral dislocation with dens fracture resulted from force to the patient's rotated head, the mechanism of injury for our case was presumed to be axial loading followed by a secondary extension force. Despite successful closed reduction, neck angio-CT scans showed some degree of residual subluxation of the C1–2 joint and type II dens fracture. Considering the severe posterior displacement of the type II dens fracture and complete rupture of the TAL, we thought that anterior dens screw fixation and cervical brace or halovest application could increase the risk of treatment failure due to dens nonunion and persistent C1–2 instability or subluxation. Therefore, we performed posterior C1–2 segmental fixation and fusion and successfully achieved anatomical reduction of C1–2 and fusion of the dens fracture.

According to the Allen classification,^[12]^ unilateral facet subluxation is considered to be stage 1 or 2 of a distractive flexion injury. In addition, fracture of posterior bony elements, including the lamina or facet joint, is generally caused by distractive extension injury. Our case was a unilateral facet fracture with subluxation and lamina fracture at C6–7. Given that the C1–2 posterolateral dislocation with dens fracture resulted from force to the patient's rotated head, the mechanism of injury for our case was presumed to be hyperextension followed by a secondary flexion injury. Treatment for unilateral facet subluxation and/or facet fracture is controversial.^[13–16]^ Nonsurgical treatment for stage 1 or 2 distractive flexion injury is reported to cause late instability in 64% of cases. In addition, facet fracture increases the risk of late instability in unilateral facet subluxation. Our patient complained of severe radiating pain to the left shoulder and arm due to unilateral facet fracture and subluxation, which caused compression of the C7 nerve root. Therefore, we performed anterior discectomy, open reduction, and fusion of C6–7 with plate fixation, and the patient's symptoms completely improved after surgery.

In conclusion, to the best of our knowledge, this is the first report of traumatic C1–2 posterolateral dislocation with dens fracture, TAL injury, and noncontiguous subaxial cervical injury. A high index of suspicion and careful evaluation of entire cervical spine should be considered as the key to the proper diagnosis and treatment of traumatic C1–2 dislocation associated with multiple contiguous and noncontiguous cervical injuries.
